# Toward a historical sociology of infectious disease governance: an interview with Alexandre White

**DOI:** 10.1590/S0104-59702025000100010

**Published:** 2025-04-07

**Authors:** Alexandre White, Gabriel Salgado Ribeiro de Sá

**Affiliations:** i Assistant Professor, Department of the History of Medicine/Johns Hopkins University. Baltimore – MD – USA, orcid.org/0000-0002-5296-6072, awhite94@jhmi.edu; ii PhD Candidate, Department of Social Sciences/Universidade Federal de Juiz de Fora. Juiz de Fora – MG – Brasil, orcid.org/0000-0001-7499-3146, gabriel.salgado@estudante.ufjf.br

**Keywords:** History of medicine, Historical sociology, Infectious disease, Epidemics, World Health Organization (WHO), História da medicina, Sociologia histórica, Doenças infecciosas, Epidemias, Organização Mundial da Saúde (OMS)

## Abstract

This interview explores Alexandre White’s contributions to the history of medicine, focusing on his latest work, *Epidemic Orientalism: race, capital, and the governance of infectious disease*, published by Stanford University Press in 2023. White’s extensive research on infectious disease regulation is examined, covering his motivations, ongoing projects, and the pivotal role of comparative methods in the field. His theoretical frameworks are also highlighted for the valuable insights they provide for understanding the complex dynamics of global health, particularly amidst emerging international tensions surrounding infectious diseases.

## Resumo

Esta entrevista explora as contribuições que Alexandre White fez para a história da medicina, com foco em seu trabalho mais recente, *Epidemic Orientalism: race, capital, and the governance of infectious disease*, publicado pela Stanford University Press em 2023. A extensa pesquisa de White sobre regulação de doenças infecciosas é abordada, examinando suas motivações, projetos em andamento e o papel crucial dos métodos comparativos em seu campo. Além disso, seus arcabouços teóricos são destacados por oferecerem *insights* valiosos para entender as dinâmicas complexas da saúde global, particularmente no contexto de tensões internacionais emergentes em torno de doenças infecciosas.

Alexandre White^1^ is a sociologist and historian of medicine with a primary research interest in comparative historical sociology. He holds the position of Assistant Professor at Johns Hopkins University in Baltimore, Maryland, USA, with affiliations in both the Department of the History of Medicine (School of Medicine) and the Department of Sociology (Krieger School of Arts and Sciences). He also serves as an Associate Director for the Center for Medical Humanities and Social Medicine at the same institution.

White’s scholarship significantly contributes to understanding racism within the context of medicine and epidemics. His work highlights how historical precedents shape contemporary responses. For instance, White (2020) argues that the xenophobia observed during the covid-19 pandemic has deep roots in nineteenth-century epidemics and early international disease control efforts. White and King (2021) also position racism within a broader historical framework, highlighting its shared ideological and practical origins linked to European modernity’s “racial project.”^2^

His main influences can be traced to three pivotal thinkers, Frantz Fanon, Stuart Hall and Edward Said, who profoundly shaped the intellectual and historical context of his work throughout his career. They are evident in three core themes that define his epistemological framework: (1) a critique of colonialism, focusing on the structures of domination and their social repercussions; (2) an examination of how power is perpetuated and reproduced through social and cultural mechanisms; and (3) a commitment to political and intellectual engagement both within and beyond academia.

White’s most recent book investigates the social impact of infectious disease outbreaks, both historically and in contemporary settings. The global mechanisms that shaped responses to these outbreaks (including how states, treaties, and institutions governed infectious diseases) are examined in detail. *Epidemic Orientalism: race, capital, and the governance of infectious disease*, published by Stanford University Press in 2023, has been praised for its significant contribution towards a “global genealogy” of infectious disease control. The historical development of international treaties and regulatory bodies since the eighteenth century was traced through extensive archival research at prestigious institutions such as the World Health Organization (WHO) archives, the Western Cape Archives, the British Library, and the Wellcome Library Archives. Insights from twelve qualitative interviews with key figures in global health also enrich this work.

The book illustrates the intricate interplay of imperialism, race, and capital in historical epidemic responses, drawing upon historical regulations as evidence to illustrate how these factors shaped responses for centuries.^3^ The broader historical, social, and economic context of epidemics is examined, analyzing how powerful nations (or empires) perceived and addressed these threats, ultimately influencing the paradigms guiding global health responses. This focus raises a compelling research question: why were international health regulations persistently prioritized for the same three diseases (plague, cholera, and yellow fever) even after the establishment of WHO and until 2005?

The key concept of “epidemic Orientalism” is introduced to address these and other questions, aligning directly with the book’s title. This term is defined as a way of perceiving and understanding infectious disease threats based on how the West historically defined itself in relation to the rest of the world (White, 2023, p.2). Building upon Edward Said’s classic concept of Orientalism (Said, 1978), this contribution involves recognizing how a dominant discourse and perspective guided the global approach to infectious diseases for centuries, stemming from Europe’s colonial interactions with the rest of the world. This framework shaped how the West perceived itself in relation to the colonized (non-European) world and the disease threats attributed to these regions (White, 2023, p.18).

The text argues that epidemic Orientalism helped create a mythology of Western supremacy concerning infectious diseases. According to this viewpoint, the West could protect itself from infectious diseases by “exiling and isolating others” (White, 2023, p.249-251). In those terms, this powerful concept encompasses both discourse (a system of knowledge production) and myth (a perpetuated and often untrue narrative) that framed the rest of the world as a perpetual source of potential epidemic outbreaks and historically served to legitimize actions taken by international players that dominated international infectious disease regulation. The text consequently maintains that the world map could be viewed in terms of not only geographical, national, or imperial boundaries, but also as a landscape of disease threat and vulnerability, defined in relation to the West (White, 2023).

This interview^4^ briefly examines the author’s work on infectious disease regulation, including his motivations, current research, and the importance of comparative methods in the field. The discussion also explores potential solutions for overcoming epidemic Orientalism within the WHO, particularly considering the changing international landscape and China’s growing influence. Special attention is given to White’s ongoing research project concerning the involvement of insurance in the transatlantic slave trade in the eighteenth and early nineteenth centuries.

*To start, I’m curious to hear about what sparked your initial interest in infectious disease regulation. Also, your research seems to consistently explore the fascinating intersection of Black studies and epidemiology. Would you be willing to elaborate on this unique aspect of your academic background?*

I’m mostly interested in how different forms of expert knowledge produce political and social subjects. So I’m curious about how medical knowledge is employed to understand conceptions of differences such as racial difference [and] cultural differences, and this comes up mostly in my book. I argue that systems of infectious disease control regulations are used to not just determine responses to epidemic threats, but to create understandings of the world that fundamentally divide the broadly Western hemisphere as the site of spaces free of infectious disease risk and sites that are interpreted as pandemic risks, that need to be protected from the rest of the world, which seem to be the perpetual potential risk sites of infectious disease. So that’s what my work explores. And, you know, my work really focuses on thinking about conceptions of risk and how risk produces subjects in the modern era.

*To what extent do you think epidemic Orientalism persists through discourse and regulatory frameworks?*

It’s visible in both, and I think there’s visibility in the regulations. It becomes most evident in the framing and drafting of these regulations. Political, economic, or social priorities

are always organized alongside epidemiological concerns. And, in this book, I explore how colonial relations prior to Second World War attempted to be maintained in post-Second World War international sanitary regulations, which evolved into the International Health Regulations. Epidemic Orientalism is about maintaining systems of relations between empires, colonies, and states in a manner that reflects their historical organization. You see it in public or media discourse as well. We can’t separate the racism and xenophobia around covid-19 from epidemic Orientalism; it’s all interconnected. I focus on how it impacts the structure of epidemic response, especially in the form of the International Health Regulations.

*Throughout your book, we observe the significant role of historical contrasts in infectious disease responses. This interest strongly resonates with some of your award-winning publications; you received two accolades from the American Sociological Association as a former graduate student. How do you think historical comparative analysis enhances our comprehension of public health crises, particularly concerning infectious diseases?*

Wow, yeah, that’s a great question. You know, in the paper you’re referencing, “Global risks, divergent pandemics” (White, 2018), I compare responses to plague and smallpox in the same city over the same time to demonstrate the force that international regulations or infectious disease were having on the plague and smallpox, and the divergent responses to two diseases, one resulting in aggressive racial segregation and quarantine. My objective was not to try to understand why these similar diseases had different outcomes, but rather to explore how concerns about them were intertwined with ideas of race and racism in Cape Town. Additionally, I examined fundamental concerns regarding isolation from trade and travel both into and out of Cape Town, as well as the various ways in which the international sanitary conventions would have restricted maritime engagement with the city. It was a very concentrated comparison. You know, the conditions were quite similar for the cases.

In the book you think a bit of a comparison, but I really wanted to provide kind of a history of, on one hand, the history of international sanitary conventions through to the International Health Regulations, and the other, I wanted to provide the history of the shifting or durable ideologies that organized these regulations, right? It’s a unique comparison, but rather, I see it as an attempt to trace the politics of knowledge that exists within global health, to name it, look at its roots, and to examine its contemporary effects. So I don’t want to say that it’s a *longue durée* history or a history of the present. I’m not just trying to explain why responses to covid happened the way they did, as a way of looking at history. Instead, I wanted to show the durability of these forms of thought from the colonial periods to the present.

I believe that comparison, when done well, is useful and effective. It highlights nuances and differences in historical contexts. I think the challenge arises when comparisons are based on nothing substantial,^5^ right? We understand the significance of smallpox compared to other contagious diseases, such as smallpox and plague. These were two diseases, two epidemics occurring simultaneously, historically treated in deeply racialized ways in South Africa or Cape Town, yet resulting in very different responses. My argument is that there was more power invested in the public health structure in Cape Town during the plague than during smallpox, along with broader international intellectual questions that also influenced the responses to these racialized situations.

*Your work emphasizes a critical point: when examining epidemics historically, the [pathogen/virus] itself isn’t the sole focus. Instead, the emphasis lies on its capacity to disproportionately impact certain regions, often the West. Given the historical dominance of Western interests in international health organizations like the WHO, how can we evolve the global health approach to prevent epidemic Orientalism from becoming the primary framework for addressing infectious diseases?*

I argue that flattening the meaning of epidemic threat to merely biological aspects erases the ways in which political, social, and economic contexts influence and impose themselves on a disease. As we saw with covid-19, the impacts of a pandemic extend beyond biology and epidemiology; they also include economic effects that intersect with capitalism in a host of ways. There are major financial crises precipitated by epidemics. Therefore, when considering the significance of infectious diseases, we must examine all these aspects. This leads us to ponder significantly about how economic and political concerns shape infectious disease control policies, often reflecting the anxieties and objectives of powerful Western states. Historically, these concerns have been evident, initially revolving around colonial and imperial anxieties. Although there have been shifts, particularly the post-Second World War and post-imperial eras, the fundamental concerns remain quite similar.

To avoid epidemic Orientalism, I believe that certain individuals within organizations like WHO are cognizant of the Orientalizing discourse interwoven with infectious disease control, whether explicitly acknowledged or not. However, addressing this issue requires a fundamental transformation of international health regulations, particularly those concerning the International Sanitary Regulations. These regulations often prioritize maximum control over infectious disease with the minimum effects to trade and traffic, reflecting a strong focus on economic interests. What if we were to think about, for instance, intellectual property sharing when it comes to vaccine access? Right, that’s a critical point. We need to change how we perceive and govern responses to infectious threats, the investment and funding of primary care and health, and health system responses across the world. This isn’t just a matter of coordination; we’ve seen that neither the US nor the British healthcare systems are equipped to manage a pandemic. Official international funding is crucial to support these efforts.

*With the rise of non-Western powers, do you believe epidemic Orientalism will eventually decline within international health regulations?*

Yes, I think it’s possible. Covid-19 exposed numerous flaws in our existing structure of infectious disease control. To mount an effective response, we must also address the epidemic Orientalism that I described. One aspect that makes Edward Said’s Orientalism concept such a useful theoretical framework for understanding the governance of infectious disease is that it wasn’t solely a Western perspective on the East. It was also a means for the West to define itself in relation to the vision it projected onto the East. By constructing an idea of the Orient for the purpose of control, they simultaneously shaped a vision of Europe as its opposite. Epidemic Orientalism similarly constructs a myth of the West as immune to disease, a stable and unified entity against the rest of the world. This perception is deeply damaging and dangerous. As I mentioned earlier, there’s a myth of modernity in the West where it’s believed to be free of disease, which is simply untrue. Do you see how detrimental this perception is, especially with leaders like Trump in the United States and Boris Johnson in the UK? It’s fundamentally unfair, not just externally, but internally as well.

*The Trump administration’s criticism of the WHO’s initial response to China raised questions about the historical power dynamics within the organization. Your work suggests that Western nations have often used the WHO for political influence. Given China’s growing financial contributions and its rise as a global power, to what extent has China challenged or influenced the historical patterns of epidemic Orientalism within the WHO, particularly in its response to the covid-19 pandemic?*

I’m not entirely sure [laughs]. I mean, I think that Trump’s outbursts towards the WHO and his threats to withdraw the United States from the organization were significant. However, I also believe that the influence of epidemic Orientalism doesn’t solely depend on the material power of the West to sustain itself. In other words, while actions like trying to withdraw from the WHO demonstrate the West’s influence, much of the power of epidemic Orientalism lies in its presence within international regulations. It’s not solely tied to the current material power of the West, but it remains a critical component in the design of international regulations. I believe change is possible, and I think it’s inevitable. As we examine the new International Health Regulations, we may witness transformations. The landscape has shifted significantly since the early twentieth century. The dominance of Western powers, like the United States and the British Empire, has waned. Even though these powers were at their peak in the 1920s, we still see remnants of epidemic Orientalism persisting through colonial and imperial decline, even after Second World War. These old colonial ways of thinking don’t simply disappear. They have the potential to endure, even without the same level of power.

*To what extent did political ideology influence Brazil’s response to covid-19, particularly regarding social distancing measures and international cooperation? Do you think Brazil aligned itself with Trump’s epidemic Orientalism discourse?*

In the chapter discussing the reform of the International Sanitary Conventions under the WHO, it becomes apparent that Brazil, at that point, was taking a stand against epidemic Orientalism, which was the dominant discourse at the time. However, in contemporary times, we have also witnessed leaders like Trump and others aligning themselves with this discourse. This appeal, especially for authoritarian leaders, stems from the notion that during times of epidemic, the problem is perceived to be caused by outsiders – an external threat. This perspective fosters the management and control of those deemed internal threats, often leading to the marginalization of minority populations. Both Trump and Bolsonaro, for instance, have expressed skepticism towards the value of vaccines. This denial, coupled with the appeal of epidemic Orientalism, serves their agenda of building a strong national state that is resistant to perceived foreign influences or individuals considered outside the realm of true citizens.

*Your book introduces the thought-provoking concept of “necrofinance,” shedding light on the troubling potential of financial markets to speculate on pandemics. This phenomenon, as you demonstrate with compelling evidence (White, 2023, p.51, 228, 243), assigns economic value to life and death, often detached from the human cost. Your ongoing research project, “Underwriting Souls,”^6^ delves further into this theme, suggesting a connection between this contemporary phenomenon and similar practices in the eighteenth and early nineteenth-century African slave trade. Could you elaborate on the continuities and/or discontinuities you observe between these historical and contemporary forms of necrofinance?*

Yes, so this is my next project. I want to explore a particular aspect of the history of capitalism, or perhaps more specifically, the history of racial capitalism, which hasn’t been adequately addressed. It’s about the phenomenon of financialization, where human life is commodified and seen as valuable to others, abstracted from the individual. It differs from traditional life insurance or other personal financial risk structures, as it involves assessing the value of individuals in both life and death. In the chapter on necrofinance, I examined the World Bank’s pandemic emergency financing facility and how it was structured around speculating on potential loss of life for the purpose of managing and providing aid. For my academic research, I aim to take a more historical approach to this subject. I want to explore similar phenomena in different contexts, particularly during early capitalism and periods of financialization. Specifically, I’m interested in examining the role of the slave trade. I’m hoping to do research back in the UK, but also probably in the Caribbean, maybe in Brazil, and elsewhere to trace this history with the hope of complicating our understanding of its origins.

*Can you tell me about Lloyd’s insurance market and their acknowledgment of their involvement in the transatlantic slave trade?*

I’ve been involved in digitizing their archive as part of an independent collaboration. We didn’t receive any funding or editorial influence from Lloyd’s. Our aim was to create an archive and exhibitions related to their archive on the Atlantic slave trade. In response, Lloyd’s provided $65 million for various initiatives aimed at addressing the negative impacts of slavery. We produced an online digital archive^7^ of these materials under a Creative Commons license. Additionally, we curated nine exhibitions exploring the history of insurance and slavery, along with a poetry and art volume titled *Uprising and resistance*. This project involved the work of nine poets and artists in the UK, aiming to confront the violence of these historical injustices.

## NOTES

1 Alexandre White holds a PhD in Sociology from Boston University, an MSc in Sociology from the London School of Economics and Political Science, and a BA in Black Studies from Amherst College. Those interested in learning more about his work can explore his personal website, which serves as a valuable resource: https://www.alexandrewhite.com/.

2 As noted by previous researchers, discussions surrounding “race,” “ethnicity,” or “color” are often fraught with controversy and paradox. Despite the lack of consensus on the exact meaning of these terms, they are frequently subject to ideological manipulation (see Munanga, 2004; Pena, 2005).

3 White’s perspective closely aligns with Zylberman’s (2020) view, emphasizing that public health, much like empires, is shaped by both internal and external forces. The interplay between the center and the periphery dictates the evolution of public health policies. These policies, in turn, impact society by implementing rules aimed at managing its outermost boundaries.

4 This interview took place in Baltimore, Maryland, United States, during our Research Trainee appointment at the Department of the History of Medicine at Johns Hopkins University.

5 While a more explicit comparison is possible in White’s study on Cape Town due to the city’s unique historical, economic, and social characteristics, in epidemic Orientalism the opposite is observed, due to the specific nature of the study of health regulations or treaties.

6 The “Underwriting Souls” project is an in-depth exploration of a disturbing aspect of history: the involvement of insurance in the transatlantic slave trade. This digital archive created by Professor Alexandre White delves into the role of Lloyd’s Insurance Market, established in 1668 by Edward Lloyd, in underwriting the financial systems that supported and prolonged the institution of slavery.

7 Access to this digital archive is available at: https://underwritingsouls.org/. Access on: 14 Apr. 2024.
